# Pico de fluxo da tosse para predizer o resultado da extubação: uma revisão sistemática e metanálise

**DOI:** 10.5935/0103-507X.20210060

**Published:** 2021

**Authors:** Natália de Araújo Ferreira, Arthur de Sá Ferreira, Fernando Silva Guimarães

**Affiliations:** 1 Programa de Pós-Graduação em Ciências da Reabilitação, Centro Universitário Augusto Motta - Rio de Janeiro (RJ), Brasil.; 2 Departamento de Fisioterapia Cardiorrespiratória e Musculoesquelética, Faculdade de Fisioterapia, Universidade Federal do Rio de Janeiro - Rio de Janeiro (RJ), Brasil.

**Keywords:** Extubação, Respiração artificial, Desmame, Tosse, Desmame da ventilação mecânica, Terapia respiratória

## Abstract

**Objetivo:**

Avaliar a utilidade do pico de fluxo da tosse para predizer o desfecho da extubação em pacientes que obtiveram sucesso no teste de respiração espontânea.

**Métodos:**

A busca cobriu as bases de dados científicos MEDLINE^®^, Lilacs, Ibecs, Cinahl, SciELO, Cochrane, Scopus, Web of Science e literatura cinzenta. Utilizaram-se os critérios Quality Assessment of Diagnostic Accuracy Studies para avaliar a qualidade da metodologia e o risco de viés dos estudos. A heterogeneidade estatística da razão de verossimilhança (LR) e razão de chance diagnóstica (RCD) do diagnóstico foram avaliadas com utilização de gráficos em floresta, teste Q de Cochran e um gráfico crosshair summary Receiver Operating Characteristic, utilizando um modelo com múltiplos pontos de corte.

**Resultados:**

Inicialmente obteve-se, nas bases de dados, um total de 3.522 referências; dentre estas, selecionaram-se para análise qualitativa 12 estudos que incluíram 1.757 participantes. Muitos estudos apresentavam um risco de viés incerto em termos da seleção de pacientes e do fluxo e tempo. Dentre os 12 estudos incluídos, sete tinham alto risco e cinco risco incerto para o item padrão de referência. O desempenho diagnóstico do pico de fluxo da tosse para o resultado da extubação foi baixo a moderado quando se consideram os resultados de todos os estudos incluídos, com +LR de 1,360 (IC95% 1,240 - 1,530), -LR de 0,218 (IC95% 0,159 - 0,293) e razão de chance diagnóstica de 6,450 (IC95% 4,490 - 9,090). Uma análise de subgrupos que incluiu somente estudos com valores de corte entre 55 e 65 L/minuto demonstrou desempenho ligeiramente melhor, porém ainda moderado.

**Conclusão:**

A avaliação do pico de fluxo da tosse, considerando valor de corte entre 55 e 65 L/minuto, pode ser útil como medida complementar antes da extubação. São necessários estudos com melhor delineamento para elucidar o melhor método e equipamento para registrar o pico de fluxo da tosse, assim como o melhor ponto de corte.

## INTRODUCTION

Mechanical ventilation (MV) is essential for interventions in respiratory failure; however, the decision about ventilatory support interruption is paramount in the care of critically ill subjects. Increases in MV duration are associated with increased mortality and complications such as ventilator-associated pneumonia, ventilator-associated lung injury, atelectasis and pneumothorax, among others.^(1^-^3)^ Therefore, discontinuation of MV should be implemented as soon as possible. On the other hand, early extubation may lead to reintubation, which also leads to increased morbidity, increased length of hospital stay and mortality.^(4, 5)^

With the goal of avoiding complications resulting from both the unnecessary presence and precocious withdrawal of the endotracheal tube, a spontaneous breathing trial (SBT) is recommended in the current weaning guidelines to assess the patient’s ability to breathe spontaneously.^(1, 6, 7)^ It can be carried out using various techniques, such as low ventilatory pressure support, continuous positive airway pressure (CPAP), automatic tube compensation, or total removal of the mechanical ventilatory support by connecting a “T”-shaped piece in the endotracheal tube to an enriched oxygen source.^(8)^ A trial is considered successful when the patient tolerates 30 minutes or more of either technique.^(6, 9)^ Although SBT has been proven to have high accuracy in predicting the weaning outcome, 12.4% to 21% of subjects who succeed in this test require reintubation within 48 to 72 hours.^(6, 7, 9^-^14)^ One of the main reasons reported for reintubation is ineffective coughing, resulting in secretion retention in the postextubation period, which cannot be predicted by the SBT.^(15^-^17)^

Many studies have reported that cough strength assessment by the measurement of cough peak flow is very accurate in predicting the extubation outcome.^(18^-^21)^ Moreover, this assessment is advocated as objective, easy to perform, inexpensive and reproducible.^(22, 23)^ Despite these promising results, there are several methodological aspects to be considered in related studies, as well as differences regarding the accuracy, the best cutoff value to predict extubation success and how to obtain the measurement. Therefore, we decided to summarize the current evidence by conducting this systematic review and meta-analysis to assess the accuracy of cough peak flow measurement, the best cutoff point, and any technical issues regarding the procedure.

## METHODS

The review methodology was defined prior to the start of data research. The protocol has been registered in the International Prospective Register of Systematic Reviews (registration number CRD42019143195). Changes were made in the initial protocol to include the possibility of further subgroup analyses. This addendum to the protocol aimed to enable the analysis of subgroups, including studies with similar characteristics, such as the use of rescue therapy, the cough stimulation method, equipment used, cutoff values, etc. However, subgroup analyses were only carried out in the presence of a sufficient number of studies with homogeneous characteristics. This systematic review was reported according to the Preferred Reporting Items for Systematic Reviews and Meta-Analyses (PRISMA) checklist.^(24)^

### Search strategy

The searches of the databases were carried out on April 3rd, 2020 guided by two experienced librarians and two researchers. They covered the following databases and portals: MEDLINE® via PubMed®; BVS Portal, including Literatura Latino-Americana e do Caribe em Ciências da Saúde (Lilacs) and Índice Bibliográfico Español en Ciencias de la Salud (Ibecs) scientific databases; Cummulative Index to Nursing and Allied Health Literature(Cinahl); Scientific Electronic Library Online (SciELO); Cochrane; Scopus; Web of Science; Embase®; and gray literature. Additionally, we searched a clinical trials registry (http://clinicaltrials.gov) for unpublished and ongoing studies.

The search strategy used the following keywords: (“artificial respiration” or “ventilation mechanical” or “intubation” or “spontaneous breathing trial” or “critical care” or “intensive care”) and (cough or “peak expiratory flow rate”) and (“airway extubation” or weaning or “ventilator weaning” or extubation). For the Embase® database, the search strategy was (“artificial ventilation” or intubation or “intensive care”) and coughing or “peak expiratory flow”) and weaning or “ventilator weaning” or “extubation failure.”

This research was designed to obtain the highest possible sensitivity, while its specificity was ensured by manual reviews of the retrieved results. FG and NA independently examined the titles and abstracts resulting from the electronic search to exclude obviously irrelevant articles. After this stage, the full texts of the other studies were evaluated. The two reviewers discussed the texts to reach a consensus when there was a disagreement.

### Inclusion and exclusion criteria

The inclusion criteria were as follows: (1) type of study: prospective or retrospective peer-reviewed studies in English, Portuguese or Spanish; (2) population: subjects older than 18 years under MV for more than 24 hours who were successful in the SBT and considered able to be extubated; (3) index test/assessment: measurement of cough peak flow prior to the extubation process; and (4) predefined results: the expected outcome of the cough peak flow assessment’s ability to predict extubation success or failure. The following were excluded: abstracts, letters, editorials, expert opinions, reviews and case reports; studies with tracheotomized subjects; and studies with subjects extubated for clinical comfort.

### Data extraction

Two reviewers extracted the data independently using a predefined data extraction form. The data extracted included the first author’s name, year of publication, study design, cough assessment method (voluntary or involuntary), instrument used for measurement, use of rescue therapy (yes or no, device), sample size (n), extubation failure or success (n), sensitivity and specificity, cutoff, area under the Receiver Operating Characteristic (ROC) curve (AUC), positive and negative predictive value, relative risk, odds ratio and positive and negative likelihood ratios. Articles by the same author were carefully examined to avoid duplication of the included studies, and any disagreement was resolved by consensus.

### Quality assessment and publication bias

The Quality Assessment of Diagnostic Accuracy Studies (QUADAS-2)^(25)^ was used to assess the methodological quality and risk of study bias. This tool is structured in four domains that present the main sources of bias, including patient selection, index testing, reference standard and flow and time. Each domain is assessed for bias risk and, except for the flow and time domains, for test applicability.

### Statistical analysis

Statistical analysis was performed by ASF using R Project version 3.6.2^(26)^ and the packages diagmeta^(27)^ and mada,^(28)^ as recommended.^(29)^

Positive and negative likelihood ratios (+LR and -LR, respectively; the magnitude by which the probability of extubating a given patient is modified by the results of the cough peak flow test) and the diagnostic odds ratio (DOR; the ratio of the odds of a positive result in a patient with successful extubation compared with a patient with unsuccessful extubation) were calculated for each study. Pooling of the indices was performed with the bivariate model of Zwindermann & Bossuyt.^(30)^ Confidence intervals (95%CIs) were calculated using Wilson’s method. The statistical heterogeneity of likelihood and DORs were evaluated using forest plots and Cochran’s Q statistic, in which each study was weighted by the use of an inverse variance model; significant heterogeneity was detected when p < 0.10 due to the number of studies included.^(31)^ To quantify the extent of heterogeneity, we used Higgins’s I^2^ statistic to measure the percentage of variability among summary indices that was caused by heterogeneity rather than chance (0% to 25%, may not be important; 25% to 50%, may represent low heterogeneity; 50% to 75%, may represent moderate heterogeneity; 75% to 100%, may represent high heterogeneity).^(31)^

The paired sensitivity and specificity values of each study are presented on a crosshair summary ROC (sROC) plot using the multiple cutoff model.^(32)^ A smoothed curve was then fitted across the studies to represent the relationship between the true positive and false positive (1-specificity) fractions of each study, from which the area under the ROC (AUROC) curve was calculated. To investigate whether variations in the diagnostic threshold affected the shape of the sROC curve, the threshold effect was tested using the regression equation log (DOR) = a + b·S, where S is a measure of the diagnostic threshold (null hypothesis: b = 0). A subgroup analysis was conducted with studies that reported a cutoff value in the range of 55 to 65L/min. Publication bias was assessed by funnel plots for the DOR using Deeks’ funnel plot asymmetry test. Significant asymmetry (p < 0.10) indicates the presence of publication bias.^(33)^

## RESULTS

### Literature search results

**Figure 1:**
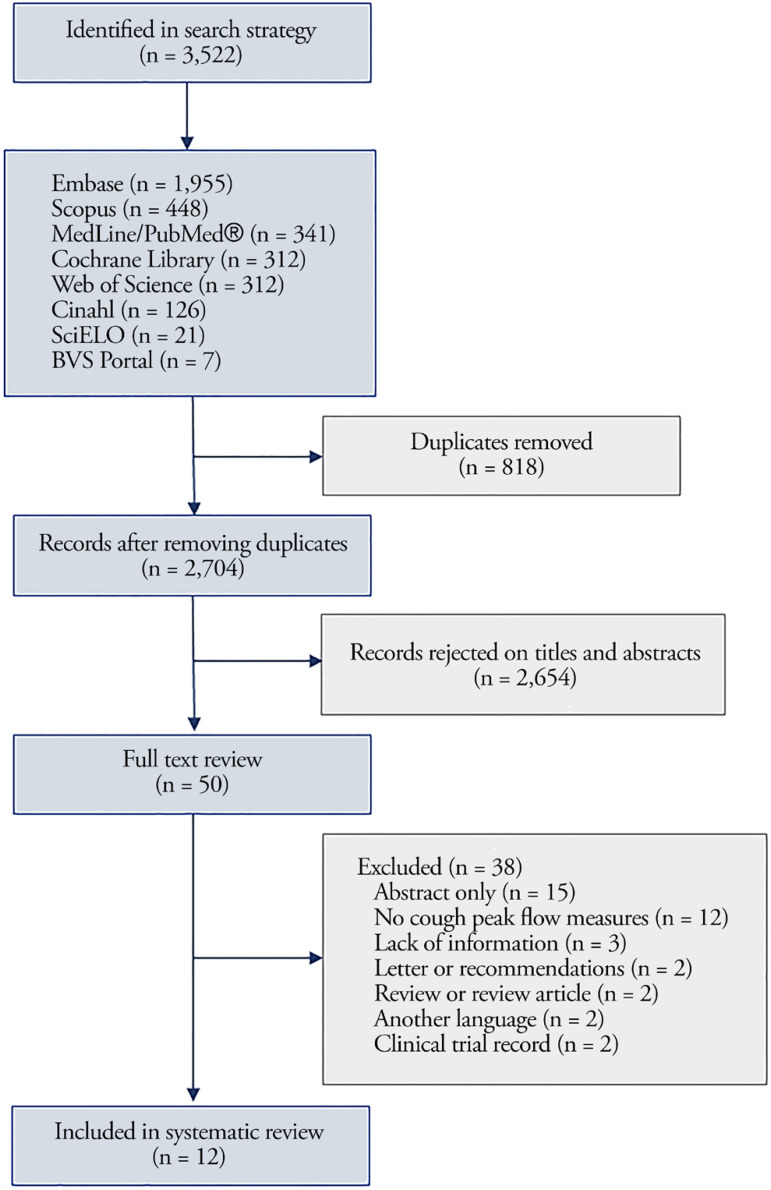
Selection of studies included in this meta-analysis. Cinahl - Cumulative Index of Nursing and Allied Health Literature; SciELO - Scientific Electronic Library Online; BVS - *Biblioteca Virtual em Saúde.*

We initially identified 3,522 references in the databases, and after the removal of 818 duplicates, we obtained 2,704 studies. Among these, we discarded 2,654 articles after reading the titles and abstracts. We examined the full texts of 50 articles and excluded 38 that did not meet the inclusion criteria. Finally, 12 articles were selected for inclusion in this review (Figure 1).

### Characteristics of the studies

The main individual characteristics of the studies are summarized in table 1. Among the 12 included studies,^(9, 18^-^20, 23, 34^-^40)^ two had their results divided for analysis.^(35, 38)^ Thus, 14 sets of results are presented in table 2, with one study published each year in 2003, 2004, 2009, 2010, 2013, 2014, 2016 and 2018, corresponding to 66%; two (17%) studies published in 2015; and two (17%) studies published in 2017. The total sample size from the included studies was 1,757 (range 88 to 356) participants, of whom 135 were classified as neurological and 125 were classified as burned. Cough assessment was performed voluntarily in nine articles and involuntarily in two articles, and one article compared the two forms. Four articles used noninvasive ventilation (NIV) as rescue therapy, and one of those also used mechanically assisted cough. Two studies assessed the cough peak flow with a mechanical ventilator, and one of those compared the evaluation of the cough peak flow between a spirometer and ventilator. The postextubation observation period was up to 72 hours in seven studies, up to 48 hours in four studies and up to hospital discharge in one study. In the studies in which there was no contingency table, we e-mailed the authors to ask for data to construct 2 x 2 tables.

The ROC curve was calculated in seven studies, while the cutoff point was defined “a priori” in five studies. The specificity and sensitivity values ranged from 55.1 to 87 and from 69 to 85, respectively. The area under the ROC curve ranged from 0.61 to 0.83, and the averaged cutoff value for the cough peak flow was 59.3 ± 9.9L/minute (range 35 to 80L/minute). Ten (71%) results from nine studies used cutoff values in the range of 56 to 62.4L/minute. The total number of successful cases was 1,677 (84%), with an average cough peak flow value of 83.6 ± 17.9L/minute (range 63.6 to 125.8L/minute). The total number of failures was 321 (16%), with an average cough peak flow value of 55.5 ± 11.1L/minute (range 36.3 to 75.8L/minute). The details of all included studies are shown in tables 1 and 2.

### Quality assessment of the included studies

**Figure 2:**
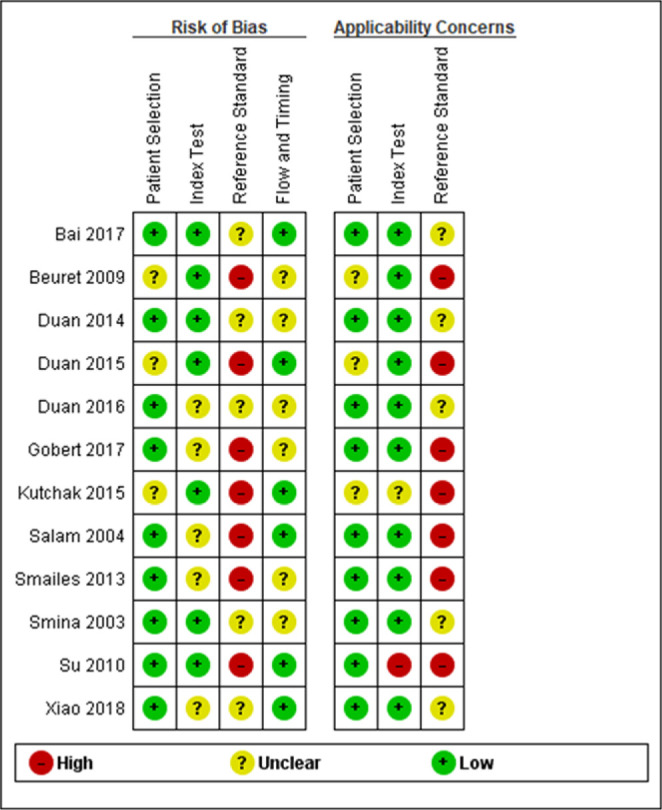
Assessment of the risk of bias of the included studies (Quality Assessment of Diagnostic Accuracy Studies 2 - QUADAS 2).

**Figure 3:**
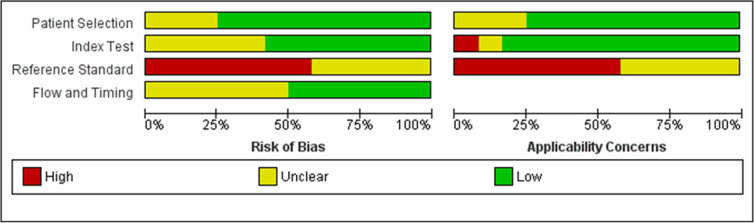
Group bar charts showing the risk of bias and applicability concerns of the twelve included records using the Quality Assessment of Diagnostic Accuracy Studies-2 (QUADAS-2).

Most studies presented a low to unclear risk of bias in the overall analysis, except for the item “reference standard.” Among the 12 included studies, seven presented a “high risk” and five an “unclear risk” due to a lack of objective clinical criteria for reintubation and the nonexclusion of subjects who were reintubated for laryngospasm. The use of NIV as rescue therapy was observed in four studies and was classified as “unclear risk” for bias in the item “flow and time”. Three studies including reintubated subjects were also assigned as having “unclear risk” in the item “flow and time.” Three studies reported that the subjects were ready to wean but did not describe specific criteria, so we assigned them to the “unclear risk” classification in the item “patient selection” (Figures 2 and 3).

### Quantitative data synthesis

**Figure 4:**
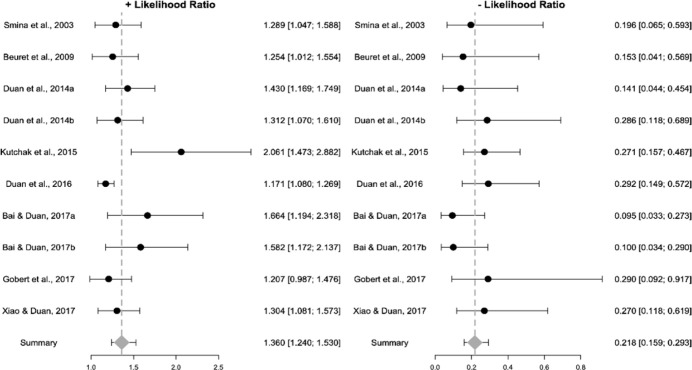
Pooled summary of positive and negative likelihood ratios of the included studies.

**Figure 5:**
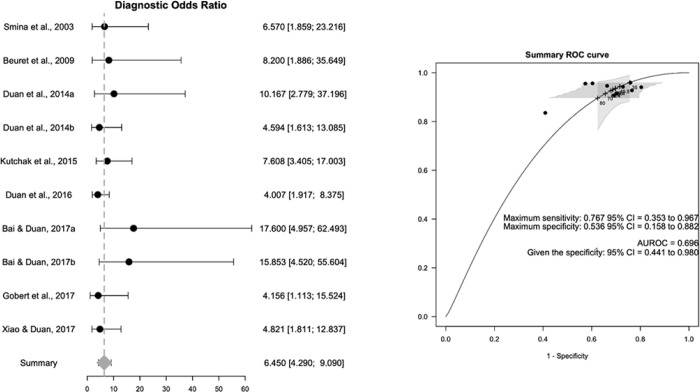
Diagnostic accuracy of the cough peak flow for the extubation outcome: pooled odds ratio and summary Receiver Operating Characteristic curve. ROC - Receiver Operating Characteristic; 95%CI - 95% confidence interval; AUROC - under Receiver Operating Characteristic.

Regarding the confusion matrix analysis, 10 (71%) results reported sensitivity and specificity values alongside the area under the ROC curve. Four (29%) results reported positive and negative predictive values. Five (36%) results reported positive likelihood values, and four (29%) reported negative likelihood values. Five (36%) studies also reported the relative risk, whereas only three (21%) reported the odds ratio. The pooled summary probabilities (Figures 4 and 5) showed that the diagnostic performance of the cough peak flow for extubation was low to moderate, with a +LR of 1.360 (95%CI 1.240 - 1.530), -LR of 0.218 (95%CI 0.159 - 0.293) and DOR of 6.450 (95%CI 4.490 - 9.090). No evidence of heterogeneity was observed for +LR (Cochran’s Q = 9.426, p = 0.399, I^2^ = 4.5%), -LR (Cochran’s Q = 7.493, p = 0.586, I^2^ = 0%) or DOR (Cochran’s Q = 7.889, p = 0.545, I^2^ = 0%).

**Table 1:** Characteristics of the included studies

Study	n	Age (years)	Duration of MV (days)	Cough assessment	Measuring instrument	Definition of extubation failure	Rescue therapy	Cutoff from ROC curve
Smailes et al.^(9)^	125	Extubation failure: 44 (27 - 70.4) Extubation success: 38 (34 - 43.6)	Extubation failure: 20 (13.3 - 28) Extubation success:6 (4 - 7)	Voluntary (coached)	Mini-Wright peak flow meter	Reintubation within 48 hours	No	No
Smina et al.^(18)^	115	Extubation failure: 62.5 ± 5.8 Extubation success: 64.1 ± 1.7	Extubation failure: 3.7 ± 0.1 Extubation success: 3.3 ± 0.2	Voluntary (coached)	Peak flow meter (nonspecified model)	Reintubation within 72 hours	No	Yes
Beuret et al.^(19)^	130	Extubation failure: 70 ± 16 Extubation success: 62 ± 17	Extubation failure: 9.5 (7 - 18) Extubation success: 8 (4 - 16)	Voluntary (coached)	Pocket spirometer (Piko-1)	Reintubation within 48 hours	Yes, NIV	Yes
Salam et al.^(20)^	88	Extubation failure: 62.0 ± 4.7 Extubation success: 62.4 ± 1.9	Extubation failure: 6.3 ± 1.9 Extubation success: 5.6 ± 0.6	Voluntary (coached)	Pneumotachograph (Aztech peak flow meter)	Reintubation within 72hours	No	No
Duan et al.^(23)^	356	CPF ≤ 70L/minute (NIV): 73 ± 12 CPF ≤ 70L/minute (Control): 74 ± 13 CPF > 70L/minute (NIV): 67 ± 14 CPF > 70L/minute (Control): 58 ± 19	CPF ≤ 70L/minute (NIV): 8 ± 12 CPF ≤ 70L/minute (Control): 7 ± 5 CPF > 70L/minute (NIV): 7 ± 5 CPF > 70L/minute (Control): 5 ± 4	Voluntary (coached)	Spirometer (Chest- graph HI-101)	Reintubation within 72hours	Yes, NIV	No
Su et al.^(34)^	150	Extubation failure: 68.0 (20.0 - 88.0) Extubation success: 66.0 (20.0 - 95.0)	Extubation failure: 4.5 (3.0 - 28.0) Extubation success: 4.0 (3.0 - 24.0)	Involuntary (saline instillation)	Hand-held respiratory mechanics monitor (nonspecified model)	Reintubation until hospital discharge	No	Yes
Duan et al.^(35)^	115	Extubation failure: 73.95 ± 15.43 Extubation success: 68.42 ± 15.12	Extubation failure: 9.58 ± 5.84 Extubation success: 5.80 ± 5.55	Voluntary and Involuntary (saline instillation)	Spirometer (Chest- graph HI-101)	Reintubation within 72hours	Yes, VNI	Yes
Duan et al.^(36)^	186	Extubation failure: 73.6 ± 14.0 Extubation success: 68.3 ± 16.0	Extubation failure: 8.6 (5.3) Extubation success: 7.4 (10.5)	Voluntary (coached)	Spirometer (Chest- graph HI-101)	Reintubation within 72hours	No	Yes
Kutchak et al.^(37)^	135	Extubation failure: 73.95 ± 15.43 Extubation success: 68.42 ± 15.12	Extubation failure: 11.46 ± 6.26 Extubation success: 7.21 ± 4.85	Involuntary (saline instillation and catheter)	Spirometer (Chest- graph HI-101)	Reintubation within 48 hours	No	Yes
Bai et al.^(38)^	126	Extubation failure: 77 ± 13 Extubation success: 66 ± 14	Extubation failure: 9.3 ± 4.4 Extubation success: 4.9 ± 4.1	Voluntary (coached)	Mechanical ventilator display (PB840, Covidien) and Spirometer (Chest- graph HI-101)	Reintubation within 72hours	No	Yes
Gobert et al.^(39)^	92	Extubation failure: 71 (65 - 78) Extubation success: 69 (60 - 75)	Extubation failure: 8 (5 - 11) Extubation success: 4 (3 - 11)	Voluntary (coached)	Built-in flow meter (hot wire technology, Spirlog, EvitaXL, Drager)	Reintubation within 48 hours	Yes, NIV and MI-E	No
Xiao et al.^(40)^	139	Extubation failure: 77 ± 11 Extubation success: 63 ± 19	Extubation failure: 9.0 ± 5.6 Extubation success: 5.7 ± 4.3	Voluntary (coached)	Spirometer (Chest- graph HI-101)	Reintubation within 72hours	No	No

MV - mechanical ventilation; ROC - Receiver Operating Characteristic; CPF - cough peak flow; NIV - noninvasive ventilation; MI-E - mechanical insufflation-exsufflation. Results expressed as mean or median (95% confidence interval), mean ± standard deviation; median (interquartile range) or median (range).

**Table 2:** Predictive power of the peak cough flow of the included studies

Study	Cutoff (L/min)	AUC (%)	Sensitivity (%)	Specificity (%)	Predictive value +	Predictive value -	Likelihood ratio +	Likelihood ratio -	Relative risk	Odds ratio
Smailes et al.^(9)^	60.0	-	-	-	-	-	-	-	9.100	1.060
Smina et al.^(18)^	60.0	70.0	69.0	74.0	-	-	-	-	5.100	-
Beuret et al.^(19)^	35.0	-	79.0	71.0	-	-	2.720	0.290	6.900	-
Salam et al.^(20)^	60.0	-	76.9	65.7	-	-	2.200	-	4.800	-
Duan et al.^(23)^	70.0	-	-	-	-	-	-	-	-	-
Su et al.^(34)^	58.5	80.2	78.8	78.1	93.0	50.0	-	-	-	0.950
Duan et al.^(35)*^	62.4	74.3	85.0	64.2	-	-	-	-	-	-
Duan et al.^(35)†^	49.8	63.2	70.0	66.3	-	-	-	-	-	-
Duan et al.^(36)^	62.4	67.8	82.1	55.1	-	-	1.830	0.320	-	-
Kutchak et al.^(37)^	80.0	-	-	-	-	-	-	-	3.600	-
Bai et al.^(38)‡^	56.4	79.0	73.0	87.0	42.3	96.0	5.430	0.310	-	-
Bai et al.^(38)§^	56.0	83.0	73.0	85.0	39.3	95.9	4.790	0.310	-	-
Gobert et al.^(39)^	60.0	61.0	70.4	63.6	93.4	22.6	-	-	-	-
Xiao et al.^(40)^	60.0	-	-	-	-	-	-	-	-	0.975

AUC - area under the curve. *Voluntary cough; †involuntary cough; ‡spirometer; §mechanical ventilation.

**Figure 6:**
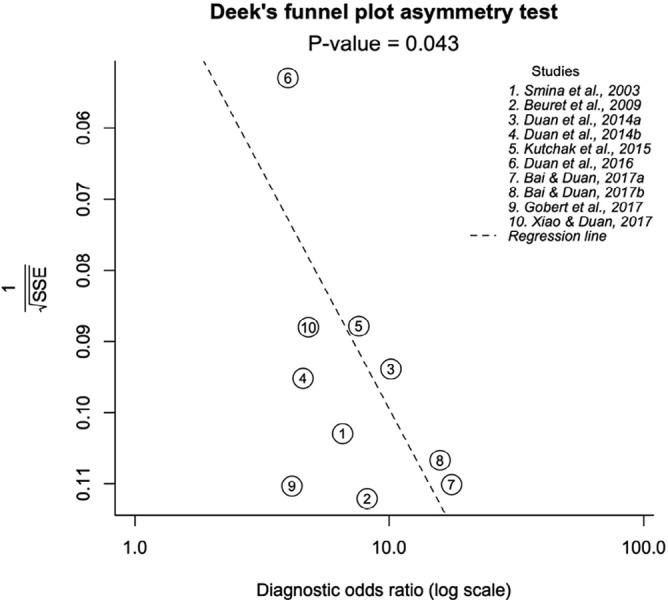
Deeks’ funnel plot asymmetry test for publication bias across studies. SSE - sample size-effectiveness. Significant asymmetry (p = 0.043) indicates the presence of publication bias.

The sROC curve yielded a maximum sensitivity of 0.767 (95%CI 0.353 - 0.967) and a specificity of 0.536 (95%CI 0.158 - 0.882) and an area under the curve of 0.696 (given sensitivity: 95%CI 0.088 to-0.015; given specificity: 95%CI 0.441 to 0.980), consistent with the moderate diagnostic accuracy of the cough peak flow for extubation (Figure 5, left panel). We observed no evidence of a threshold effect (b = -0.007, p = 0.668). Figure 6 shows Deeks’ funnel plot asymmetry test for publication bias. We observed a significant asymmetry (p = 0.043), indicating the presence of publication bias across studies.

### Subgroup analysis

Six (43%) studies reported a cutoff value in the range of 55 to 65L/minute. The pooled summary probabilities showed that the diagnostic performance of the cough peak flow for extubation was slightly higher than the overall quantitative synthesis, with a +LR of 1.390 (95%CI 1.270 - 1.540), -LR of 0.176 (95%CI 0.109 - 0.267), and DOR of 8.400 (95%CI 4.740 - 13.600). No evidence of heterogeneity was observed for +LR (Cochran’s Q = 4.417, p = 0.491, I^2^ = 0%), -LR (Cochran’s Q = 4.339, p = 0.501, I^2^ = 0%), or DOR (Cochran’s Q = 4.827, p = 0.437, I^2^ = 0%). Due to the limited number of studies, no subgroup analysis was conducted for the sROC curve. Likewise, we observed no evidence of a threshold effect (b = -0.155, p = 0.182).

## DISCUSSION

The current recommendations for extubation readiness testing are focused on SBT performance; however, it is known that this trial cannot assess individuals’ capacity to protect their airways, which is directly related to the extubation outcome. Therefore, considering that extubation failure is associated with increased mortality and MV stay among ICU subjects,^(1, 16)^ a number of authors have assessed the cough strength in subjects who succeed in an SBT, with the goal of predicting the extubation outcome.

The evaluation of cough peak flow is an objective method to predict successful extubation, which can be carried out voluntarily or involuntarily. Most of the studies in this review evaluated voluntary coughing, which depends on the patient’s motivation, effective coordination and the preservation of respiratory neuromuscular activity. Two studies evaluated the subjects in an involuntary way - i.e., one triggered by the cough reflex.^(34, 37)^ Su et al. considered this method more natural, being similar to the physiological cough and having the advantage of covering uncooperative subjects who do not want or are unable to produce a cough voluntarily.^(34)^ Duan et al., in a study with 115 subjects (5 of whom were noncooperative), compared voluntary and involuntary cough assessments (by instilling 2mL of saline), concluding that voluntary coughing is not invasive and has a greater capacity to predict extubation failure than involuntary coughing in cooperative subjects.^(35)^ As the cough reflex response may be directly proportional to the stimulation, it is not known whether a stronger stimulus, such as mechanical stimulation with a catheter or instilling a higher saline volume, would produce better results. Nevertheless, the instillation of saline solution could be uncomfortable and could lead to transitory desaturation. Thus, voluntary measures would be more suitable for cooperative individuals, whereas involuntary stimulation could be reserved for noncooperative subjects. Another alternative is using voluntary testing first in cooperative subjects. If the cough peak flow is below the success threshold, an effective involuntary stimulation would be applied to discard false-negative results due to the low motivation of the subjects.

Most of the studies used an external spirometer to record the cough peak flow. However, two studies used mechanical ventilator displays. Bai et al. concluded that both methods had good predictive accuracy for reintubation (AUC = 0.79 for the spirometer *versus* AUC = 0.83 for the ventilator, p = 0.26).^(38)^ Similarly, Gobert et al. used a flow meter built into a ventilator, showing good predictive capacity (AUC = 0.61) and highlighting the advantage of not requiring additional costs to purchase a device or having to disconnect the patient.^(39)^ Although a measurement using the mechanical ventilator is more practical, some aspects must be taken into account, such as how much the ventilator circuit resistance reduces the cough peak flow (perhaps determining a lower cutoff), what frequency response and sampling frequency are needed in the ventilator acquisition system and what the most appropriate ventilatory parameters are necessary to perform the measurement. Other technical aspects can also affect the reliability of the cough peak flow measurements, regardless of whether they are made with a ventilator or with an external device. The European Respiratory Society (ERS)^(41)^ carried out a complete—although not specifically designed for the target population of our study—update on respiratory muscle tests since its last recommendations in 2002, advising on the importance of the standardization of peak expiratory flow measurements. Aspects such as the number of maneuvers, patient position and possible discrepancies between different measurement instruments were pointed to as important contributors to different results across the studies. The authors of the articles selected for this review used different measurement instruments, and generally, they did not describe how the maneuvers were performed. The position of the subjects, level of consciousness, endotracheal tube size, number of measurements and the interval(s) between them, acceptability criteria for the measurements, and how the researchers performed the verbal stimulation are examples of data not reported in most studies.

Among the studies included in this review, Beuret et al. presented the lowest cough peak flow cutoff value at 35L/minute, which may be considered an outlier compared to the other values.^(19)^ This difference could be attributed to the abovementioned issues and the lack of information about how long the subjects remained in an SBT, since a longer time might have predisposed subjects to fatigue prior to the assessment, resulting in lower cough peak flow values. Moreover, the use of NIV as rescue therapy may have postponed the reintubation of subjects with a low cough peak flow, causing them to be reintubated beyond the 48 hours that determined extubation failure in their study. At the other extreme, the cutoff value of 80L/min found in the study of Kutchak et al. may also be an outlier compared to the values of the other studies in this review.^(37)^ Some factors that may have contributed to this result include subjects who were younger than in the other studies and mostly male and the use of the Mini Wright flowmeter, which may have resulted in overreading and measurement bias.^(42, 43)^

When we analyzed the risk of bias, the “reference standard” criterion presented “high risk” or “unclear risk” in most studies. For this review, the reference standard was considered the outcome of extubation—that is, as essential to calculating the predictive power of cough peak flow. Unfortunately, seven^(9, 19, 20, 34, 36, 37, 39)^ of the 12 included studies did not report the criteria that guided decision-making regarding reintubation. Moreover, the authors did not exclude reintubations due to laryngospasm and laryngeal edema, which are relatively common causes of extubation failure and are not related to the subjects’ ability to eliminate secretions. Another possible source of bias was the difference in the temporal criterion to define extubation failure. Some authors considered reintubation within 48 hours, while others considered 72 hours after extubation as the failure criterion.

The inclusion of subjects who had already been extubated was scored as “unclear risk” in the “flow and time” criterion,^(9, 18, 35)^ since reintubation is associated with a longer duration of intensive care and hospital stay, an increased incidence of ventilator-associated pneumonia, laryngeal edema and increased mortality.^(44, 45)^ Although the evidence is scarce regarding the use of NIV to prevent reintubation,^(45)^ four studies^(19, 23, 35, 39)^ in this review have included this procedure as rescue therapy. Even when NIV is not effective at preventing reintubation, its use may delay it. Therefore, as the “reference standard” uses a 48- or 72-hour time criterion, we considered that the use of NIV during the postextubation period represents an “unclear” risk of bias in the “flow and time” criterion. In three studies,^(19, 36, 37)^ “unclear risk” was given in the “patient selection” criterion. These studies did not sufficiently describe the parameters for considering subjects ready to wean, the criteria for interrupting the SBT or that subjects had to pass the SBT to be included. Studies that did not sufficiently describe how the cough peak flow measurement was performed or that established the cutoff a priori (and not from the ROC curve) also received an “unclear” or “high risk” rating in the “index test” criterion.

Despite the methodological limitations, different measurement instruments and ways in which the cough peak flow was measured, we observed that nine of the 12 studies in this review had cough peak flow cutoff values between 56 and 62.4L/minute, presenting high sensitivity and specificity to predict success in planned extubation.^(9, 18, 20, 34^-^36, 38^-^40)^ Among these nine studies, five calculated the cutoff from the ROC curve.^(18, 34^-^36, 38)^ Because these results strongly suggest that the best cutoff to predict the outcome of extubation is approximately 60L/minute, some authors adopted this threshold when determining the cutoff a priori in their studies.^(9, 20, 39, 40)^ No study considered the factors of age, sex or endotracheal tube size (endotracheal tubes with smaller diameters may determine lower cough peak flow results for the same individuals), which might be confounders for determining the best cutoff. Age and sex are directly related to the predicted peak expiratory flow. Therefore, it is likely that women and older people may have a cough peak flow that is lower but within a normal range. Moreover, table 1 shows that in practically all included studies, the mean age was higher among patients who failed extubation. Considering that older individuals have a greater risk of failure and lower predicted peak flow values, collinearity may have occurred in predicting the extubation outcome. Thus, future studies should control for the factors age, sex and endotracheal tube diameter to assess the peak cough flow’s predictive power concerning the extubation outcome.

When we analyzed the subgroup of studies with cutoff values between 55 and 65L/minute, the diagnostic performance of the cough peak flow was slightly higher than the overall quantitative synthesis, reinforcing the current assumption that the best cutoff is approximately 60L/minute.

The rationale of cough peak flow as a predictor of extubation outcome in subjects who succeed in an SBT is based on secretion retention during the postextubation period due to cough ineffectiveness. Therefore, measures should be taken to optimize airway clearance and to prevent reintubation in subjects who present with a low cough peak flow. Some evidence reinforces this premise, such as a study from Duan et al., who divided their sample of 356 individuals who succeeded in an SBT into subjects eligible for treatment with NIV or conventional oxygen therapy (control group).^(23)^ Their results showed that for subjects with a cough peak flow ≤ 70L/minute, NIV reduced reintubation compared to the control group (9% *versus* 35% postextubation up to 72 hours, p < 0.01). Subjects with cough peak flow > 70L/minute did not benefit from NIV, which strengthens the hypothesis that NIV as rescue therapy may have been a source of bias in another three cough peak flow studies included in this review.^(19, 35, 39)^

As limitations of this review, we found that the included studies used different methods, rescue therapies, extubation failure criteria, populations, and devices to perform the measurements. These differences, the lack of relevant information, and some other methodological limitations of the included studies make it difficult to devise recommendations for recording the cough peak flow and the best cutoff point associated with the extubation outcome. Moreover, along with a possible presence of publication bias, all of these abovementioned issues may have contributed to the asymmetry observed in the funnel plot analysis (Figure 6)^(46)^ and the moderate diagnostic performance found in the statistical analysis.

## CONCLUSION

The cough peak flow recording is promising for improving approaches to subjects during the process of mechanical ventilation withdrawal. The studies included in this review make it very clear that reduced cough peak flow values are associated with extubation failure. The cutoff of approximately 60L/minute seems to have the best accuracy. However, recommendations about how to perform the measurement are necessary so that well-designed studies using standardized protocols can in the future determine the best cutoff associated with the extubation outcome.
